# Retrospective comparison of myopia progression between low-concentration atropine combined with spectacles and spectacles alone in myopic children aged 6 to 10 years

**DOI:** 10.1097/MD.0000000000047770

**Published:** 2026-03-13

**Authors:** Zhen Ning, Xiaojuan Rong, Yunxia Ding, Yingqing Xiang, Liqiang Ge

**Affiliations:** a Nanchang Key Laboratory of Glycolipid Metabolism and Chronic Diseases, Jiangxi institute of Technology, Nanchang City, Jiangxi Province, China; b General Internal Medicine Department, Jiangxi Provincial Children’s Hospital, Nanchang City, Jiangxi Province, China.

**Keywords:** accommodative function, axial length, childhood myopia, low-concentration atropine, safety, spectacles

## Abstract

This study aimed to evaluate the efficacy and safety of low-concentration atropine combined with spectacles in controlling myopia progression among school-aged children and to assess treatment differences across age and refractive subgroups. This retrospective controlled study included 80 myopic children (160 eyes) who visited our center between January 2022 and January 2024. Among them, 40 patients (80 eyes) received low-concentration atropine combined with spectacles (combination group), and 40 patients (80 eyes) used spectacles alone (control group). All participants completed baseline, 6-, 12-, and 18-month follow-ups. Changes in spherical equivalent (SE), axial length, visual function (including best-corrected visual acuity and amplitude of accommodation) and adverse events were compared between groups. Subgroup analyses were conducted according to age and baseline refractive error. Statistical analyses included *t* tests, chi-square tests, and repeated-measures analysis of variance. Baseline demographic and refractive parameters did not differ significantly between the 2 groups (*P* > .05). After 18 months, myopia progression was significantly slower in the combination group compared with the control group (–0.45 ± 0.33 D vs –1.00 ± 0.39 D, *P* < .001), with an annual progression rate of approximately –0.30 D/year. Axial elongation was also significantly reduced (0.19 ± 0.14 mm vs 0.36 ± 0.17 mm, *P* < .001), and SE change was positively correlated with axial growth (*R* = 0.70, *P* < .001). Best-corrected visual acuity remained stable in both groups, while amplitude of accommodation increased significantly in the combination group (*P* = .03). Mild and reversible adverse reactions occurred in 10.0% of the combination group, primarily transient photophobia and near blur, with no serious events reported. Subgroup analysis revealed greater efficacy in younger children aged 6 to 8 years and those with baseline SE ≤ –2.00 D (*P* < .05). Low-concentration atropine combined with spectacles effectively slows myopia progression and axial elongation in children aged 6 to 10 years while maintaining stable visual function and demonstrating good safety and tolerability. The combined intervention provides superior control compared to optical correction alone, particularly benefiting younger and early-onset myopic children. These findings offer new clinical evidence supporting the combined application of low-concentration atropine in pediatric myopia management.

## 1. Introduction

In recent years, the prevalence of myopia among children and adolescents has increased dramatically worldwide, reaching epidemic levels in East Asian countries.^[1,2]^ Epidemiological data indicate that the overall prevalence of myopia among Chinese school children exceeds 50%, and in urban areas it is even above 70%. Moreover, the onset age has shifted younger, with the 6- to 10-year-old period identified as a critical window for both the onset and rapid progression of myopia.^[3,4]^ Early-onset myopia in children is associated with a faster rate of progression and a higher risk of developing high myopia in later life, which in turn predisposes to sight-threatening complications such as retinal degeneration, myopic maculopathy, and optic atrophy.^[5]^ Therefore, delaying myopia progression and controlling axial elongation have become central goals in ophthalmic research and public health interventions.

The pathogenesis of childhood myopia is multifactorial, involving the complex interplay between genetic predisposition and environmental influences.^[6,7]^ While heredity determines the baseline susceptibility, environmental factors (particularly excessive near work and insufficient outdoor activity) are recognized as the major external drivers of the current myopia epidemic.^[8,9]^ Modern children spend prolonged periods using electronic screens under intense academic demands, leading to sustained accommodative stress, ciliary muscle spasm, scleral remodeling, and subsequent axial elongation, ultimately resulting in progressive refractive shifts.^[10]^ Conventional optical interventions, such as single-vision spectacles or orthokeratology lenses, primarily act by improving retinal image quality or modulating peripheral defocus to slow myopia progression.^[11,12]^ However, these methods address optical consequences rather than the underlying biological mechanisms of eye growth, and thus their efficacy in halting axial elongation remains limited.

Against this backdrop, low-concentration atropine eye drops have emerged as a pharmacologic approach with promising outcomes in myopia control. Atropine, a nonselective muscarinic receptor antagonist, inhibits cholinergic signaling in the retina and sclera, thereby modulating ocular growth and delaying myopia progression.^[13,14]^ Early studies demonstrated that high-concentration atropine (0.5–1.0%) markedly slows myopia progression but causes significant side effects, including photophobia, mydriasis, and near-vision blur, which limit its long-term use.^[15,16]^ In contrast, low concentrations (0.01–0.05%) maintain substantial efficacy with far fewer adverse effects, showing excellent safety and tolerability. Multiple randomized controlled trials have confirmed that low-dose atropine effectively reduces both myopic refractive changes and axial elongation by approximately 40% to 60%, with minimal rebound after discontinuation.^[17,18]^ Nevertheless, heterogeneity remains regarding optimal dosage, treatment duration, and response in different age groups, and evidence among younger children remains relatively limited.

Spectacles remain the most widely used and accessible means of vision correction. While they improve visual acuity and optical quality, their impact on controlling myopia progression is limited and may even increase accommodative demand, potentially accelerating axial elongation.^[19,20]^ Recently, a “combined intervention” strategy (integrating pharmacologic and optical approaches) has attracted growing attention. Low-concentration atropine may biochemically suppress ocular growth, whereas spectacles provide stable retinal imaging and reduce accommodative stress. These complementary mechanisms may yield synergistic benefits, maintaining visual performance while providing structural protection. In clinical practice, this combined approach is also highly feasible, given the ease of atropine administration and the widespread use of spectacles, making it a safe, convenient, and adherent option for pediatric myopia management.

Although numerous studies have examined low-concentration atropine monotherapy, systematic evaluations of its combined use with standard spectacle correction remain scarce. Most previous investigations have focused on older school-aged children, whereas evidence for younger children aged 6 to 10 years, a critical period of rapid ocular growth, is insufficient. Furthermore, treatment response to atropine appears to vary by age and baseline refractive status, and some studies suggest that earlier intervention yields better outcomes, though this has not been comprehensively validated in combination therapy models.^[21,22]^ Therefore, exploring the long-term efficacy and safety of low-concentration atropine combined with spectacles across different age and refractive subgroups holds both theoretical and clinical significance.

Based on this rationale, the present study retrospectively compared low-concentration atropine combined with spectacles versus spectacles alone in controlling myopia progression, axial elongation, and visual function among children aged 6 to 10 years. Subgroup analyses were further conducted to examine differential efficacy according to age and baseline refractive error. This research aimed to evaluate the real-world effectiveness and safety of the combined regimen under routine clinical conditions. The innovation of this study lies in 3 aspects: it uses real-world clinical data, reflecting outcomes in practical, non-experimental settings; it incorporates multidimensional efficacy indicators, including refractive, structural, and functional parameters, to provide a more comprehensive assessment; and it applies subgroup analyses to identify variability across different populations, offering insights for personalized myopia control strategies.

In summary, low-concentration atropine combined with spectacle correction may represent a safe, effective, and scalable approach for integrated myopia management in children. By comparing its outcomes with conventional optical correction and analyzing subgroup-specific responses, this study provides novel clinical evidence and new perspectives for optimizing pediatric myopia prevention and individualized intervention.

## 2. Methods

### 2.1. Study design and participants

This study was approved by the Ethics Committee of Jiangxi Provincial Children’s Hospital. This study was designed as a retrospective controlled trial and included 80 children with myopia (160 eyes) who attended the ophthalmology outpatient clinic of our hospital between January 2022 and January 2024. Participants were divided into 2 groups according to parental preference and treatment regimen: a low-concentration atropine (Hunan Wuzhoutong Pharmaceutical Co., Ltd., National Medicine Permit Number H43020903) combined with spectacles group (combination group, n = 40, 80 eyes) and a spectacles-only group (control group, n = 40, 80 eyes). All subjects were diagnosed with mild-to-moderate myopia and were aged between 6 and 10 years. None had strabismus, amblyopia, or structural ocular abnormalities such as corneal or lenticular pathology. The study protocol was approved by the Institutional Ethics Committee and adhered to the tenets of the Declaration of Helsinki. Written informed consent was obtained from the legal guardians of all participants prior to inclusion.

### 2.2. Inclusion and exclusion criteria

Inclusion criteria were as follows: age between 6 and 10 years; spherical equivalent (SE) refractive error ranging from –1.00 D to –3.50 D in both eyes; confirmed true myopia after cycloplegic refraction; and ability to comply with regular follow-up examinations. Exclusion criteria included: prior treatment with orthokeratology lenses or atropine; presence of amblyopia, strabismus, fundus pathology, or other media abnormalities; systemic diseases or long-term medication use that could affect accommodative function or myopia progression; and poor compliance or incomplete clinical data.

### 2.3. Ophthalmic examinations and measurement procedures

All examinations were performed by the same team of certified ophthalmologists and optometrists. Refractive error (SE) was measured 3 times under cycloplegia using an autorefractometer, and the average value was recorded. Axial length (AL) was measured using optical biometry (IOL Master 700, Carl Zeiss, Germany). Corneal curvature (K) was automatically calculated by the same device. Best-corrected visual acuity (BCVA) was recorded in logarithm of the minimum angle of resolution units. The amplitude of accommodation (AA) was assessed 3 times using a near-point ruler, and the mean value was used for analysis.

Physical parameters, including height, weight, systolic blood pressure (SBP), diastolic blood pressure (DBP), and heart rate (HR), were recorded along with parental history of myopia and comorbidities. All measurements were performed in the morning under stable illumination to minimize circadian influences on ocular biometry.

### 2.4. Treatment and follow-up protocol

Participants in the combination group received 0.01% atropine eye drops once daily at bedtime, 1 drop per eye, continuously for 18 months. The control group wore spectacles for distance correction without pharmacologic intervention. All participants underwent follow-up examinations at baseline, 6, 12, and 18 months, including assessments of BCVA, SE, AL, and AA. Adverse events were systematically recorded by the research team, including symptom type, duration, and severity. If participants experienced significant discomfort or decreased vision, atropine was temporarily discontinued and reevaluated by the attending ophthalmologist to determine whether treatment should be resumed.

### 2.5. Statistical analysis

All data were analyzed using SPSS version 26.0 (IBM Corp., Armonk). Continuous variables were expressed as mean ± standard deviation. Between-group comparisons were conducted using the independent samples *t* test, while categorical variables were compared using the χ^2^ test or Fisher exact test when appropriate. Repeated-measures analysis of variance (repeated-measures ANOVA) was applied to evaluate time effects, group effects, and interaction effects. Pearson correlation analysis was used to assess associations between continuous variables. A 2-tailed *P* value of <.05 was considered statistically significant.

## 3. Results

### 3.1. Baseline characteristics of participants

Between January 2022 and January 2024, a total of 80 children with myopia (160 eyes) who met the inclusion criteria were enrolled in this study. Among them, 40 cases (80 eyes) received low-concentration atropine in combination with spectacle correction (combination group), and 40 cases (80 eyes) received spectacle correction alone (control group). All participants completed baseline examinations and initial follow-up assessments, with no missing key variables. There were no statistically significant differences between the 2 groups in terms of sex distribution, age, height, weight, SBP, DBP, HR, or primary refractive parameters, including SE refraction, axial length (AL), and corneal curvature (*K*) (all *P* > .05). Moreover, there were no significant differences between groups in parental myopia history or comorbidities, indicating good baseline comparability of physiological and ocular characteristics.

Overall, the mean age of the enrolled children was 8.3 ± 1.1 years, with boys slightly outnumbering girls (53.8% vs 46.2%). The mean height and weight were approximately 130 cm and 28.7 kg, respectively. Baseline vital signs (SBP, DBP, and HR) were within the normal physiological range for children of similar age, suggesting an overall healthy study population. Regarding refractive characteristics, baseline SE ranged from –1.00 D to –3.50 D, with an average of –2.12 ± 0.80 D, consistent with mild-to-moderate myopia. The mean AL was approximately 24.1 mm, and the mean corneal curvature (*K*) was 43.2 D. Family history analysis revealed that 68.8% (55/80) of children had at least 1 myopic parent. Additionally, 12.5% (10/80) of children presented with mild comorbidities, such as allergic rhinitis, dry eye, or mild anisometropia, without any severe systemic diseases. In summary, there were no significant differences between the combination and control groups regarding baseline demographic, systemic, or refractive parameters (*P* > .05), indicating well-balanced baseline characteristics and ensuring the reliability of subsequent efficacy comparisons. Details are presented in Table 1.

**Table 1:** Baseline characteristics of participants (mean ± SD).

Variable	Combination group (n = 40)	Control group (n = 40)	*t*/χ^2^ value	*P* value
Age (yr)	8.3 ± 1.1	8.2 ± 1.0	0.44	.66
Sex (male/female)	22/ 18	21/ 19	0.05	.83
Height (cm)	130.0 ± 6.3	129.4 ± 6.5	0.56	.58
Weight (kg)	28.8 ± 4.6	28.5 ± 4.3	0.36	.72
Systolic blood pressure (mm Hg)	104.7 ± 7.6	104.1 ± 7.9	0.40	.69
Diastolic blood pressure (mm Hg)	65.2 ± 6.3	65.6 ± 6.5	0.29	.77
Heart rate (beats/min)	85.6 ± 7.2	85.9 ± 7.4	0.24	.81
Baseline spherical equivalent (SE, D)	−2.14 ± 0.79	−2.10 ± 0.82	0.35	.73
Axial length (AL, mm)	24.11 ± 0.51	24.08 ± 0.50	0.50	.62
Mean corneal curvature (K, D)	43.24 ± 1.07	43.17 ± 1.10	0.47	.64
Parental myopia [n, (%)]	27 (67.5%)	28 (70.0%)	0.05	.80
Comorbidities [n, (%)]	5 (12.5%)	5 (12.5%)	0.00	1.00

SD = standard deviation.

### 3.2. Comparison of myopia progression rate

**Figure 1.**
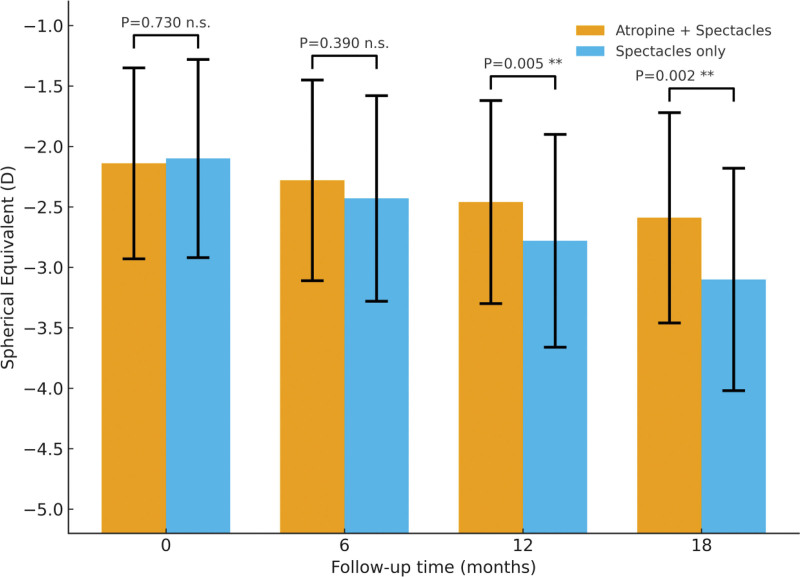
Between-group comparison of absolute spherical equivalent across 18 months.

During the 18-month follow-up period, all 80 participants (160 eyes) completed refraction examinations at baseline, 6, 12, and 18 months. Both groups showed a progressive increase in myopic refractive error over time; however, the progression rate in the low-concentration atropine combined with spectacle correction group (combination group) was significantly slower than that in the spectacle-only group (control group). From baseline to 18 months, the mean change in SE was –0.45 ± 0.33 D in the combination group and –1.00 ± 0.39 D in the control group, with a highly significant between-group difference (*t* = 7.12, *P* < .001). When converted to the annual progression rate, the combination group exhibited an average of approximately –0.30 D/year, compared with –0.67 D/year in the control group. Additionally, 62.5% (50/80 eyes) of eyes in the combination group showed myopia progression less than –0.75 D, while only 32.5% (26/80 eyes) in the control group met this criterion (χ^2^ = 10.7, *P* = .001). Repeated-measures ANOVA revealed significant main effects of group (*F* = 28.6, *P* < .001) and time (*F* = 65.4, *P* < .001), as well as a significant group × time interaction (*F* = 8.1, *P* = .005). These findings indicate that the combination treatment consistently slowed myopia progression throughout the 18-month follow-up period, with a sustained and time-dependent therapeutic benefit. Detailed results are presented in Table 2 and Figure 1.

**Table 2:** Comparison of myopia progression between the 2 groups (mean ± SD).

Follow-up time	Combination group (n = 40) SE (D)	Control group (n = 40) SE (D)	*t* value	*P* value
Baseline	−2.14 ± 0.79	−2.10 ± 0.82	0.35	.73
6 mo	−2.28 ± 0.83	−2.43 ± 0.85	0.86	.39
12 mo	−2.46 ± 0.84	−2.78 ± 0.88	2.93	.005
18 mo	−2.59 ± 0.87	−3.10 ± 0.92	3.23	.002
Total myopia progression (baseline –18 mo, D)	−0.45 ± 0.33	−1.00 ± 0.39	7.12	<.001
Myopia increase <−0.75 D [n, (%)]	50/80 (62.5%)	26/80 (32.5%)	χ^2^ = 10.7	.001

SD = standard deviation, SE = spherical equivalent.

### 3.3. Axial length changes

During the 18-month follow-up period, both groups exhibited progressive elongation of AL; however, the increase was significantly slower in the combination group (low-concentration atropine plus spectacle correction) compared with the control group (spectacles only). From baseline to 18 months, the cumulative AL elongation was 0.19 ± 0.14 mm in the combination group versus 0.36 ± 0.17 mm in the control group, showing a statistically significant difference (*t* = 5.02, *P* < .001). At specific time points, AL elongation at 12 months was 0.13 ± 0.11 mm in the combination group and 0.26 ± 0.15 mm in the control group (*P* = .001), with the difference further widening at 18 months. Repeated-measures ANOVA revealed significant main effects of group (*F* = 26.8, *P* < .001) and time (*F* = 112.5, *P* < .001), as well as a significant group × time interaction (*F* = 7.6, *P* = .007), indicating that the combined treatment produced a sustained inhibitory effect on axial elongation throughout the follow-up period. Furthermore, correlation analysis demonstrated a significant positive association between axial elongation over 18 months and the degree of myopic refractive progression (ΔSE) (*R* = 0.70, *P* < .001), suggesting that greater axial elongation was closely linked to faster myopia progression. Detailed results are presented in Table 3.

**Table 3:** Comparison of axial length changes between the 2 groups (mean ± SD, mm).

Follow-up time (mo)	Combined group (n = 40) ΔAL (mm)	Control group (n = 40) ΔAL (mm)	*t* value	*P* value
6	0.07 ± 0.08	0.13 ± 0.10	2.88	.005
12	0.13 ± 0.11	0.26 ± 0.15	3.51	.001
18	0.19 ± 0.14	0.36 ± 0.17	5.02	<.001
Baseline AL (mm)	24.11 ± 0.51	24.08 ± 0.50	0.50	.62
AL at 18 mo (mm)	24.30 ± 0.55	24.44 ± 0.57	1.20	.23

AL = axial length, SD = standard deviation.

### 3.4. Visual function changes

**Figure 2.**
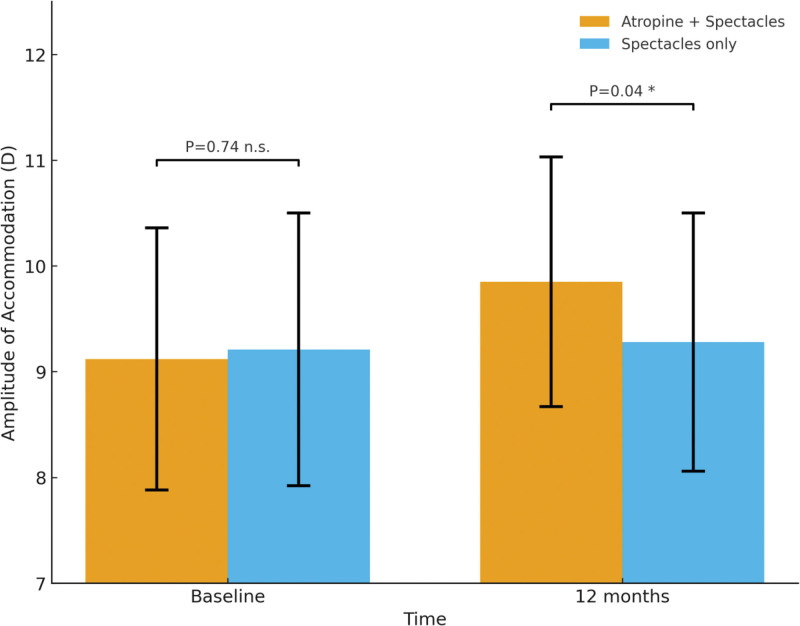
Changes in amplitude of accommodation (AA, D) at baseline and 12 months.

Throughout the follow-up period, BCVA (logarithm of the minimum angle of resolution) remained stable in both groups, with no statistically significant differences observed either within or between groups (*P* > .05). This finding suggests that low-concentration atropine had no adverse effect on distance visual acuity. Regarding AA, a significant improvement was observed in the combination group compared with baseline, increasing from 9.12 ± 1.24 D to 9.85 ± 1.18 D (*P* = .03). In contrast, the control group showed no significant change (9.21 ± 1.29 D → 9.28 ± 1.22 D, *P* = .72). Between-group comparison at 12 months indicated that the AA of the combination group was significantly higher than that of the control group (*P* = .04). These results suggest that low-concentration atropine not only maintains stable distance visual acuity but may also improve accommodative function in some children, possibly by alleviating accommodative spasm and enhancing near focusing capacity. Detailed results are shown in Table 4 and Figure 2.

**Table 4:** Comparison of visual function between the 2 groups (mean ± SD).

Parameter	Time	Combined group (n = 40)	Control group (n = 40)	Between-group *P* value
BCVA (LogMAR)	Baseline	0.04 ± 0.06	0.05 ± 0.05	.58
	12 mo	0.03 ± 0.05	0.04 ± 0.06	.61
Amplitude of accommodation (AA, D)	Baseline	9.12 ± 1.24	9.21 ± 1.29	.74
	12 mo	9.85 ± 1.18	9.28 ± 1.22	.04
	Within-group change (*P*)	0.03	0.72	–

BCVA = best-corrected visual acuity, LogMAR = logarithm of the minimum angle of resolution.

### 3.5. Adverse reactions and safety

During the entire treatment and follow-up period, 6 cases (10.0%) of mild adverse reactions were reported in the combination group, including photophobia (3 cases, 5.0%), near blur (2 cases, 3.3%), and mild conjunctival hyperemia (1 case, 1.7%). All adverse reactions were mild, transient, and resolved spontaneously or after temporary discontinuation of medication without the need for treatment cessation. No moderate or severe adverse events, such as allergic reactions or persistent visual deterioration, were observed. In contrast, no adverse reactions were reported in the control group. The overall incidence of adverse events did not differ significantly between groups (χ^2^ = 1.16, *P* = .28). Overall, low-concentration atropine demonstrated good tolerability and high safety, with no significant systemic or ocular complications detected throughout the study period. Detailed data are presented in Table 5.

**Table 5:** Comparison of adverse events between the 2 groups.

Type of adverse event	Combined group (n = 40)	Control group (n = 40)	*P* value
Photophobia	3 (7.5%)	0 (0%)	–
Near blur	2 (5.0%)	0 (0%)	–
Mild conjunctival hyperemia	1 (2.5%)	0 (0%)	–
Any adverse event	6 (15.0%)	0 (0%)	.28
Serious adverse event	0	0	–

### 3.6. Subgroup analysis

**Figure 3.**
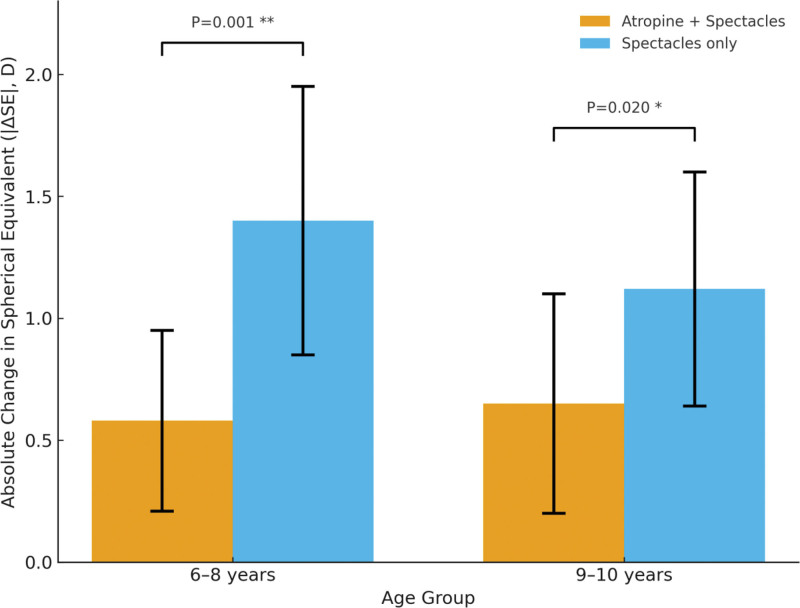
Subgroup analysis of myopia progression according to age.

To further investigate the differences in therapeutic efficacy of low-concentration atropine among different subpopulations, subgroup analyses were conducted based on age and baseline refractive error. Results showed that in the 6- to 8-year-old group, myopia progression was significantly slower in the combination group compared with the control group, with 24-month SE changes of –0.58 ± 0.37 D vs –1.40 ± 0.55 D (*P* < .001). In the 9- to 10-year-old group, SE changes were –0.65 ± 0.45 D for the combination group and –1.12 ± 0.48 D for the control group, and although the difference remained statistically significant, the magnitude of effect was smaller (*P* = .02). These findings indicate that the myopia-inhibitory effect of low-concentration atropine is more pronounced in younger children. Further analysis stratified by baseline refractive error revealed that children with higher initial myopia (SE ≤ –2.00 D) achieved better myopia control compared with those with SE > –2.00 D. Specifically, after 24 months, SE progression was –0.59 ± 0.38 D vs –1.25 ± 0.50 D (*P* = .01). This suggests that early initiation of atropine intervention yields greater efficacy, particularly in younger or more myopic children. Detailed data are presented in Table 6 and Figure 3.

**Table 6:** Subgroup analysis of myopia progression (24 months, mean ± SD, D).

Subgroup	Combined group ΔSE (D)	Control group ΔSE (D)	*P* value
6–8 yr	–0.58 ± 0.37	–1.40 ± 0.55	<.001
9–10 yr	–0.65 ± 0.45	–1.12 ± 0.48	.02
SE ≤ –2.00 D	–0.59 ± 0.38	–1.25 ± 0.50	.01
SE > –2.00 D	–0.64 ± 0.44	–1.05 ± 0.47	.07

SE = spherical equivalent.

## 4. Discussion

In recent years, the prevalence of myopia among children and adolescents has increased dramatically worldwide, with an earlier onset and faster progression, posing a major public health concern that threatens visual health.^[23]^ Controlling myopia progression and slowing axial elongation have become global research priorities. Numerous studies have demonstrated that low-concentration atropine can effectively inhibit myopia progression, while spectacle lenses remain the most common optical correction method for improving distance vision. However, optical correction alone has limited efficacy in controlling myopia development.^[24,25]^

The present retrospective comparative study investigated the efficacy and safety of low-concentration atropine combined with spectacle correction in school-aged children and further explored treatment differences across age and refractive subgroups. Unlike previous trials, this study was conducted under real-world clinical follow-up conditions and incorporated both structural and functional parameters, providing more comprehensive evidence for pediatric myopia control.

At baseline, the 2 groups were well balanced in demographics and ocular parameters, ensuring comparability and reliability of the outcomes. The results demonstrated that low-concentration atropine combined with spectacles significantly slowed myopia progression. After 18 months of follow-up, the mean refractive change was –0.45 D in the combination group versus –1.00 D in the control group (*P* < .001). Compared with previous studies using atropine monotherapy, our findings indicate that the combination therapy achieved comparable inhibition with greater long-term stability. The underlying mechanism may involve atropine blocking the retinal–scleral signaling pathway to suppress posterior ocular growth, while spectacles provide optical correction that reduces accommodative myopic drive. The synergistic effect of both interventions may better align with the multifactorial regulation of myopia development.

Axial elongation reflects the structural essence of myopia progression. In this study, axial elongation was significantly slower in the combination group (0.19 mm) than in the control group (0.36 mm) over 18 months (*P* < .001). Moreover, axial elongation was positively correlated with refractive progression (*R* = 0.70, *P* < .001), suggesting that structural suppression through pharmacologic intervention was consistent with refractive improvement. The stronger inhibition of axial growth observed in this study compared with previous reports of atropine monotherapy may be attributed to stable retinal imaging and reduced near-focus demand provided by spectacle correction. This dual mechanism may help limit excessive axial stretching, mitigate posterior segment remodeling, and potentially prevent pathological myopia over the long term.

Functionally, low-concentration atropine did not adversely affect distance vision. The BCVA remained stable throughout follow-up in both groups, consistent with previous domestic and international clinical studies confirming the safety of low-dose atropine in maintaining visual function. Interestingly, a mild improvement in AA was observed in the combination group, suggesting that prolonged use of low-dose atropine may not weaken accommodation but rather improve near focusing ability in some children. This could be related to a reduction in ciliary muscle tension and relief of accommodative spasm, thereby alleviating muscular strain associated with prolonged near work.

Treatment safety is a key consideration for long-term pediatric medication use. During follow-up, only 6 mild and reversible adverse events (10.0%) were reported in the combination group, including transient photophobia, mild near blur, and conjunctival hyperemia, all of which resolved spontaneously or after temporary discontinuation. No moderate or severe adverse events such as allergic reactions or persistent vision loss occurred. These findings are consistent with international studies reporting similar safety profiles, confirming that 0.01% atropine is well tolerated and safe for long-term use in children.^[26,27]^ Notably, no persistent pupil dilation, glare, or visual deterioration was observed, suggesting low systemic and ocular risk during extended treatment.

Subgroup analysis revealed that the therapeutic benefit was greater in younger children (6–8 years) than in older ones (9–10 years), and that children with higher baseline myopia (SE ≤ –2.00 D) achieved superior control compared to those with milder myopia. This finding aligns with the “early intervention enhances efficacy” hypothesis, suggesting that initiating atropine therapy during the early phase of myopia progression can yield better outcomes. Younger children are more responsive to environmental and pharmacologic modulation due to rapid ocular development, allowing early intervention to effectively inhibit axial elongation. Similarly, children with lower myopia may exhibit higher visual plasticity and reduced accommodative stress, facilitating greater treatment responsiveness. These results underscore the importance of age- and refractive status-specific management strategies for individualized myopia control.

The synergistic mechanism of the combined regimen may involve both biochemical and optical pathways. Low-concentration atropine suppresses axial elongation by blocking muscarinic signaling in the retina and sclera, thereby modulating ocular growth at the biochemical level. Meanwhile, precise spectacle correction improves retinal image clarity and reduces optical blur, which may otherwise generate hyperopic defocus signals that stimulate further axial elongation. The combination of these mechanisms may jointly enhance the inhibitory effect on myopia progression.

This study has several strengths. First, it utilized a real-world retrospective design based on consecutive clinical cases, reflecting treatment efficacy under routine practice conditions and improving external validity. Second, the extended 18-month follow-up provided valuable insights into the sustained effects of combination therapy beyond the typical 12-month observation period. Third, by integrating refractive, structural, functional, and safety indicators, the study offered a multidimensional evaluation of efficacy. In addition, the inclusion of subgroup analyses enriched evidence on population-specific responsiveness to low-concentration atropine. Because treatment allocation was based on parental preference rather than randomization, potential selection bias may exist. Parents opting for atropine may differ from those choosing spectacles alone in terms of myopia awareness, lifestyle habits, or compliance, which could influence myopia progression independently of treatment. Although baseline characteristics were comparable, unmeasured confounders may still affect the interpretation of the reported treatment effects.

This study has several limitations. First, its retrospective design and parental preference-based treatment allocation introduce potential selection bias, which may influence treatment outcomes despite comparable baseline characteristics. Second, analyses were conducted based on individual eyes, which may underestimate inter-eye correlations and slightly inflate statistical power. Third, retrospective data collection carries inherent risks of incomplete or missing information, which may affect the accuracy of longitudinal comparisons. Fourth, unmeasured confounding factors such as outdoor activity, near-work duration, and digital screen exposure could not be fully controlled and may have contributed to differences in myopia progression. These limitations should be considered when interpreting the results, and future prospective randomized controlled trials are needed to validate our findings.

In conclusion, low-concentration atropine combined with spectacle correction significantly slows myopia progression and axial elongation in children aged 6 to 10 years, while maintaining stable visual function and exhibiting high safety and tolerability. Its mechanism may involve reduced accommodative load, modulation of retinal signaling, and delayed scleral remodeling. Combined pharmacologic–optical intervention appears superior to optical correction alone, particularly in early-onset myopia. Future multicenter, large-sample prospective studies are warranted to validate these findings and explore optimal combinations of atropine concentrations and optical interventions, thereby providing stronger scientific evidence for pediatric myopia prevention and control.

## 5. Conclusion

Low-concentration atropine combined with spectacle correction exerts a significant inhibitory effect on myopia progression in school-aged children. This combined approach effectively slows both refractive deterioration and axial elongation while maintaining stable distance visual acuity, demonstrating high safety and tolerability. The results of this study indicate that, compared with optical correction alone, combination therapy provides a more stable and sustained suppression of myopia progression. The underlying mechanism may involve reduced accommodative demand, modulation of the retinal–scleral signaling pathway, and inhibition of posterior ocular growth.

Furthermore, younger children and those with higher baseline myopia showed greater therapeutic benefit, suggesting that early intervention can substantially enhance treatment efficacy. Adverse reactions to low-concentration atropine were mild, transient, and fully reversible, with no severe ocular or systemic complications observed, supporting its long-term safety and feasibility.

In summary, low-concentration atropine combined with spectacle correction represents a safe, effective, and practical strategy for controlling myopia in children, particularly those in the early stages of disease. Future multicenter, large-scale prospective studies are warranted to further optimize drug concentration and combination protocols, thereby providing stronger evidence for evidence-based myopia prevention and control in the pediatric population.

## Author contributions

**Conceptualization:** Zhen Ning, Xiaojuan Rong, Yunxia Ding, Yingqing Xiang, Liqiang Ge.

**Data curation:** Zhen Ning, Xiaojuan Rong, Yunxia Ding, Yingqing Xiang, Liqiang Ge.

**Formal analysis:** Zhen Ning, Xiaojuan Rong, Yunxia Ding, Yingqing Xiang, Liqiang Ge.

**Funding acquisition:** Yunxia Ding, Yingqing Xiang, Liqiang Ge.

**Investigation:** Liqiang Ge.

**Writing – original draft:** Zhen Ning, Xiaojuan Rong, Liqiang Ge.

**Writing – review & editing:** Zhen Ning, Xiaojuan Rong, Liqiang Ge.
